# Recognize fish as food in policy discourse and development funding

**DOI:** 10.1007/s13280-020-01451-4

**Published:** 2021-01-16

**Authors:** Abigail Bennett, Xavier Basurto, John Virdin, Xinyan Lin, Samantha J. Betances, Martin D. Smith, Edward H. Allison, Barbara A. Best, Kelly D. Brownell, Lisa M. Campbell, Christopher D. Golden, Elizabeth Havice, Christina C. Hicks, Peter J. Jacques, Kristin Kleisner, Niels Lindquist, Rafaella Lobo, Grant D. Murray, Michelle Nowlin, Pawan G. Patil, Douglas N. Rader, Stephen E. Roady, Shakuntala H. Thilsted, Sarah Zoubek

**Affiliations:** 1 Michigan State University, 1405 S. Harrison Road, Room 115, East Lansing, MI 48823 USA; 2 Nicholas School of the Environment, Duke University, 135 Duke Marine Lab Rd., Beaufort, NC 28516 USA; 3 Duke University’s Nicholas Institute for Environmental Policy Solutions, P.O. Box 90335, Durham, NC 27708 USA; 4 Duke University Marine Lab, 135 Duke Marine Lab Rd, Beaufort, NC 28516 USA; 5 Michigan State University, 1405 S. Harrison Road, Room 318, East Lansing, MI 48823 USA; 6 Nicholas School of the Environment, Duke University, Box 90328, Durham, NC 27708 USA; 7 WorldFish, Jalan Batu Maung, Batu Maung, Bayan Lepas, Penang 11960 Malaysia; 8 U.S. Agency for International Development, 907 Westwood Drive, NE, Vienna, VA 22180 USA; 9 Sanford School of Public Policy, Duke University, 201 Science Drive, Campus, Box 90245, Durham, NC 27708 USA; 10 Harvard T.H. Chan School of Public Health, 665 Huntington Ave. Bldg. 2, Boston, MA 02115 USA; 11 Department of Geography CB#3220, University of North Carolina, Chapel Hill, 220 Carolina Hall, Chapel Hill, NC 27599-3220 USA; 12 Lancaster Environment Centre, Lancaster University, Lancaster, LA1 4YQ UK; 13 University of Central Florida, 4297 Andromeda Loop N. Howard Phillips Hall, Rm. 302, Orlando, FL 32816-1356 USA; 14 Environmental Defense Fund, 18 Tremont Street, Ste. 850, Boston, MA 02108 USA; 15 UNC Institute of Marine Sciences, 3431 Arendell Street, Morehead City, NC 28557 USA; 16 Duke Law School, 210 Science Drive, Durham, NC 27708 USA; 17 The World Bank Group, 1818 H Street N.W., Washington, DC 20433 USA; 18 Environmental Defense Fund, 4000 Westchase Blvd., Suite 510, Raleigh, NC 27607 USA; 19 Duke Nicholas School of the Environment, Duke University, 1201 Pennsylvania Ave NW, Washington, DC 20004 USA; 20 World Food Policy Center, Duke University, 1201 Pennsylvania Avenue NW, Suite 500, Washington, DC 20004 USA

**Keywords:** Aquaculture, Fish, Fisheries, Food and nutrition security, International development, Policy

## Abstract

The international development community is off-track from meeting targets for alleviating global malnutrition. Meanwhile, there is growing consensus across scientific disciplines that fish plays a crucial role in food and nutrition security. However, this ‘fish as food’ perspective has yet to translate into policy and development funding priorities. We argue that the traditional framing of fish as a natural resource emphasizes economic development and biodiversity conservation objectives, whereas situating fish within a food systems perspective can lead to innovative policies and investments that promote nutrition-sensitive and socially equitable capture fisheries and aquaculture. This paper highlights four pillars of research needs and policy directions toward this end. Ultimately, recognizing and working to enhance the role of fish in alleviating hunger and malnutrition can provide an additional long-term development incentive, beyond revenue generation and biodiversity conservation, for governments, international development organizations, and society more broadly to invest in the sustainability of capture fisheries and aquaculture.

**Electronic supplementary material:**

The online version of this article (10.1007/s13280-020-01451-4) contains supplementary material, which is available to authorized users.

## Introduction

The global sustainable development community is off track from meeting international targets for hunger and malnutrition (an abnormal physiological condition caused by inadequate, unbalanced, or excessive consumption of macro- and/or micronutrients). If the current upward trend continues, the number of undernourished people in the world is predicted to increase from 678 million in 2018 to 841 million by 2030, figures that do not yet account for the effects of the COVID-19 pandemic, which could result in an additional 83 to 132 million undernourished people in 2020 (FAO et al. 2020). While childhood stunting prevalence has begun to decrease, the rate of decline is insufficient to achieve the desired 50% reduction by 2030; meanwhile, rates of obesity are rising in all regions of the world (FAO et al. 2020). The causes of malnutrition are multifaceted, but access to diverse, nutritious, safe, and affordable food is crucial to addressing the problem.

As countries make commitments toward achieving international targets, such as those defined in the 2015 UN Sustainable Development Goals (SDGs), a growing body of research indicates the need for policies that enhance the role of fish^1^ in achieving food and nutrition security^2^ (HLPE 2014; Béné et al. 2015; Bennett et al. 2018; Tlusty et al. 2019). Yet, because discussions of fish and food and nutrition security have traditionally been disconnected from one another (Béné et al. 2015), fish food systems fall short of their full potential to enhance food and nutrition security for those most in need. Furthermore, under-recognition of fish as a key source of nutrition misses an opportunity to promote and justify investments in improving fisheries governance and responsible fishing.

Creating policies that support the food and nutrition security contributions of fish—both wild caught and farmed—will require food, fisheries, and aquaculture policy discourses to reframe fish *as food*, a subtle but meaningful departure from the dominant paradigm of fish as a natural resource. Here, we outline the importance of fish to food and nutrition security. We then provide evidence that international efforts to achieve food and nutrition security under-represent fish and, at the same time, that capture fisheries and aquaculture policy dialogues are disconnected from objectives of nourishing the world. To help address this challenge, we identify four key pillars of research needs and policy directions that would emerge from a ‘fish as food’ global policy dialogue and enhance the role of fish in achieving food and nutrition security.

## Fish can play a crucial role in achieving global food and nutrition security

**Fig. 1:**
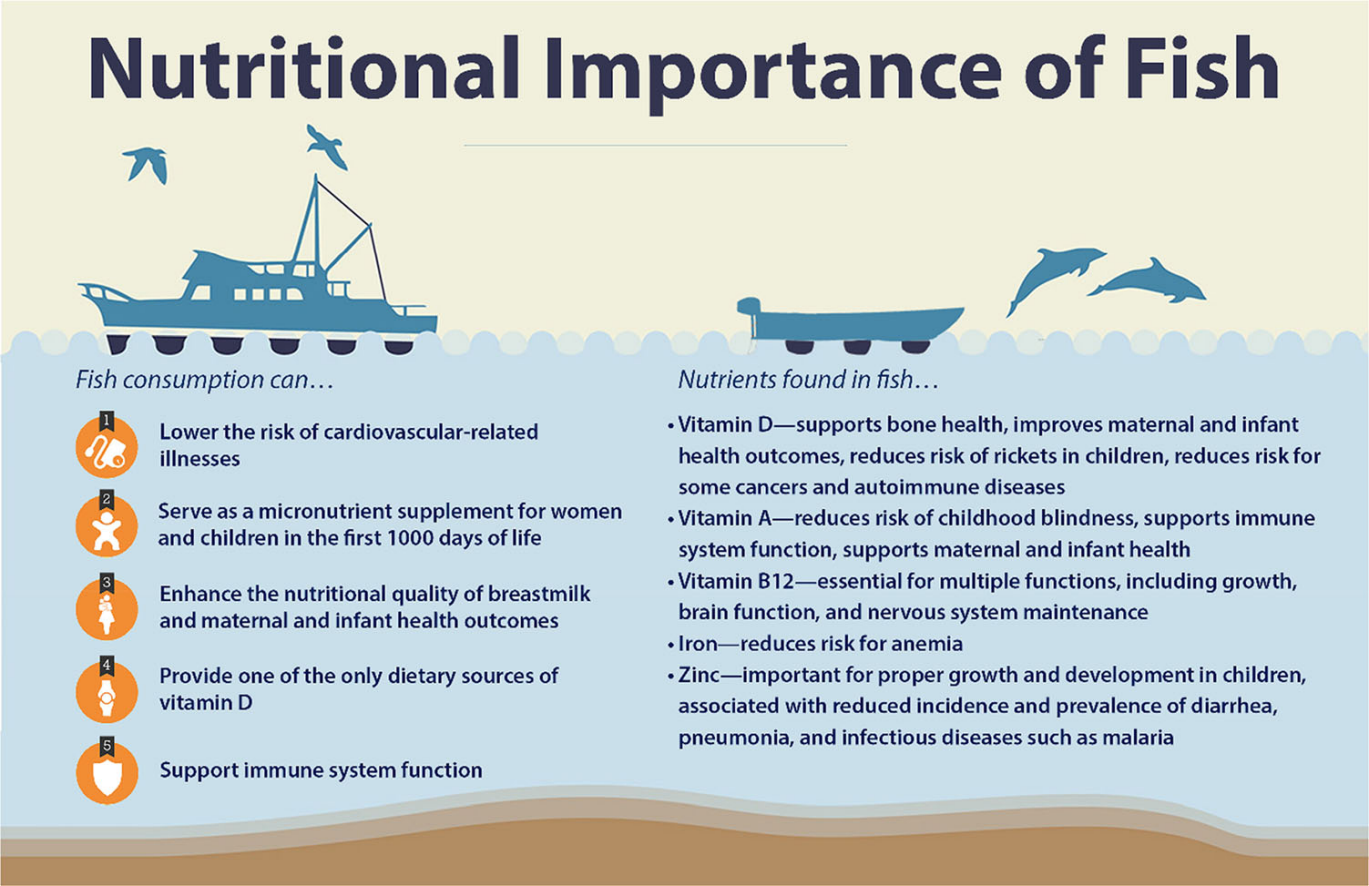
The nutritional importance of fish (Bennett et al. 2018)

Fish is an animal-source food (ASF), rich in micronutrients, essential fatty acids, and animal protein, which can help support cognitive development, alleviate stunting, improve maternal and childhood health outcomes, strengthen the immune system, and reduce cardiovascular disease (Fig. 1, Thilsted et al. 2016). Fish provide 17% of animal protein and 7% of total protein consumed globally (FAO 2020). ASF (including fish) consumption is associated with reduced childhood stunting due to higher concentrations and bioavailability of key micronutrients compared to plant-source foods (Headey et al. 2018). Additionally, fish high in essential fatty acids can reduce risks for cardiovascular disease, with 1.4 million cardiovascular-related deaths worldwide in 2010 attributable to diets low in fish-source omega-3 fatty acids (Lim et al. 2012). Thus, fish nutrients can alleviate conditions related to undernutrition as well as non-communicable disease risk.

In many contexts, fish is more affordable than other ASFs such as red meat (Funge‐Smith and Bennett 2019), making it more accessible to the poor, although prices for some species are driven up by global demand. Nine countries—all from the Global South—obtain at least half of their animal protein from fish.^3^ As a wild food, fish is often available to landless people who cannot produce crops and serves as a safety net for people during economic and climate-driven shocks and geopolitical conflicts affecting land-based food production (Funge-Smith and Bennett 2019). In some regions, aquaculture prices have fallen over time, demonstrating that farming fish has the potential to serve as a pro-poor food production system (Edwards et al. 2019). Besides being affordable and accessible, fish production systems often provide crucial nutrients with less detrimental environmental impact than other ASFs (Gephart et al. 2016; Hilborn et al. 2018).

## The fish-food disconnect in policy and development funding priorities

Fish is largely missing from key food policy dialogues and associated funding. The targets for the second SDG (SDG 2—Zero Hunger) define aims for agricultural systems that are supposed to drive policy reforms and funding; for example, resilient agricultural practices, land and soil quality, plant and livestock gene banks, agricultural subsidies, and access to land.^4^ Yet, SDG 2 targets do not mention fisheries or aquaculture by name, nor do they offer specific guidance relevant to fish production systems. The absence of fish is also noticeable in the annual Global Nutrition Report, a mechanism for tracking the commitments made by 100 stakeholders spanning governments, aid donors, civil society, the United Nations, and businesses, which mentioned fish for the first time in 2017.^5^

**Fig. 2:**
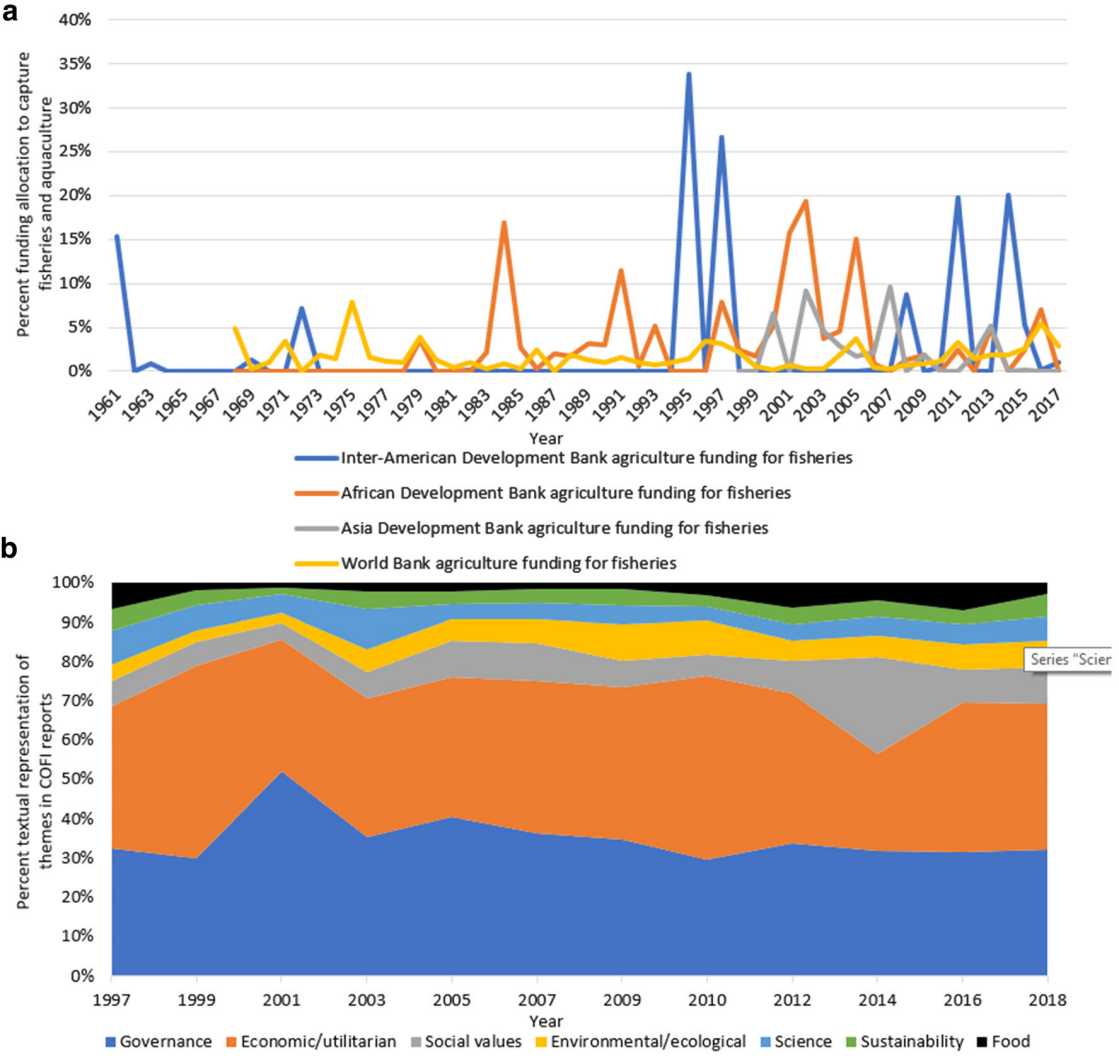
Representation of fish in development funding priorities and policy discourse. **a** World Bank and Regional Development Bank agriculture funding allocation to capture fisheries and aquaculture. Note *y* axis extends only to 40%. **b** Representation of food and other themes in Committee on Fisheries reports

Fish also appears underrepresented in international development funding priorities. For example, World Bank funding targeting capture fisheries and aquaculture averaged about 1.8% of total funding allocated to agriculture from 1968 to 2018, although over the last decade the average has been higher—about 2.6% (up to 5.4% in 2018). The Regional Development Banks have allocated a slightly higher average percentage of funding to capture fisheries and aquaculture than the World Bank over this same time period, but in many years did not fund any capture fisheries or aquaculture projects (Fig. 2, Electronic supplementary material). While this funding allocation aligns roughly with fish’s contribution to total global energy intake, energy provided is a poor measure of the importance of fish. More crucially, fish as an ASF provides highly bioavailable essential micronutrients and fatty acids, especially in low-income countries located at low latitudes (Hicks et al. 2019). Furthermore, funded projects focus on economic development rather than food and nutrition security objectives, with unclear impacts for nutritionally vulnerable people. The Bill and Melinda Gates Foundation, the world’s largest private foundation, has also historically left out fish despite a focus on global nutrition. The Foundation’s Annual Letters, published since 2009 to document their funding aims and rationale, have never mentioned fish, seafood, fisheries, or aquaculture, while referring to agriculture, farming, and crops more than 100 times. Notably, however, the Foundation began to scope fish-related projects in 2017.^6^ While fish is largely missing from funding priorities, discussions about *food* are also scarce in high-level fisheries policy. Although fish is primarily harvested for food (88% of fish harvested is currently destined for direct human consumption (FAO 2020), the framing of fish as a natural resource dominates in many key international policy arenas. For example, since 1997, the Committee on Fisheries (COFI), the forum of FAO member countries which convenes every two years to discuss the global fisheries agenda, has focused primarily on economic dimensions and only marginally on fish as food (Fig. 2, Electronic supplementary material). It was not until 2012 that fish was included in FAO’s Food Price Index, an important instrument for tracking and predicting food crises (Tveterås et al. 2012). Even SDG 14 (Life Under Water) addresses failures to sustainably manage fisheries largely through a conservation lens, emphasizing marine protected areas, ocean acidification, pollution, and the economic contributions of ocean resources, rather than using a food provisioning lens. Notably, SDG 14 excludes freshwater fisheries and inland aquaculture entirely.

This type of paradigmatic framing is important because high-level capture fisheries and aquaculture policy discussions and funding investments in food systems shape the kinds of policies that are conceivable and achievable (Dryzek 2013). For example, framing fish only as a natural resource focuses policy on species management and conservation, profits, and high-value export-oriented fisheries. While these are important, they lead to an under-emphasis of the nutritional characteristics, accessibility, seasonality, distribution, equity, and patterns of fish consumption. Focusing on fish exclusively as a natural resource presumes that management need only to attend to the economic and biological health of fish production sectors, providing little guidance on how policies contribute to or impede the attainment of food and nutrition security.

## Four pillars of “fish as food” research needs and policy directions

Framing fish as food can stimulate innovative policies and actions that support the role of fish in contributing to global food and nutrition security. The four pillars below suggest actions to move from framing fish as food towards guiding policy and investments.

### Improve metrics

Policy and funding decisions are constrained by the paucity of metrics to assess and communicate the contributions of fish to food and nutrition security. Furthermore, data gaps and weak measurement systems can undermine the achievement of development targets and objectives once they are established (Jacob 2017). Few fish species have been evaluated for their micro- and macronutrient compositions, limiting global statistics to figures on total catch volumes and estimates of protein and fat supply. Furthermore, national statistics generally underreport small-scale and subsistence fish production, as limited resources are allocated to track revenue-generating capture fisheries. However, improving assessment and governance of subsistence and low-revenue fisheries may be economically—as well as socially—meaningful, given the high economic returns on investments from reducing childhood stunting (Hoddinott et al. 2013). Emerging data sets are improving assessment of the micronutrient contributions of fish production systems. For example, the GENuS database^7^ has begun to collate and combine micronutrient content with food production figures, but source data remain scarce and piecemeal. This database enabled predictions that declines in marine fish catch over the next three decades, due to poor management and environmental factors, could subject 845 million people (11% of the world’s population) to vitamin A, zinc, or iron deficiencies (Golden et al. 2016). Recent modeling approaches are beginning to fill gaps in nutrient profiles (Hicks et al. 2019). Thus, governments and researchers can work together to develop innovative, cost-effective assessment tools to improve tracking of small-holder production and subsistence harvests, the corresponding provision of nutrients (especially micronutrients) and include these data in national and regional food composition tables (Bogard et al. 2015). This knowledge is crucial to raising the profile of fish in broader food and nutrition security policies and investment priorities.

### Promote nutrition-sensitive fish food systems

From agricultural systems, we know that implementing policies to improve the nutrition sensitivity of food systems requires pushing back against entrenched political and economic interests in food value chains to prioritize nutrients over inexpensive energy (Pinstrup-Andersen 2013). Fisheries and aquaculture policies and investments must follow suit. Modifying species composition and/or feed composition in aquaculture systems can target specific micronutrient deficiencies and optimize nutritional yield (Bogard et al. 2017). Just as strong investment enabled the development of the biofortified^8^ orange sweet potato (well-known for helping to address vitamin A deficiency), investments in fish-based solutions can contribute to solving micronutrient deficiencies. For example, the small, low-cost fish, mola (*Amblypharyngodon mola*), from the Gangetic floodplains, which can be easily produced in homestead ponds, contains more than 2500 RAE (retinol activity equivalent) vitamin A per 100 g raw, edible portion (more than twice that contained in orange sweet potato) (Hotz et al. 2012; HLPE 2014). In capture fisheries, managing for optimal nutritional yield likely requires policy measures distinct from those targeting maximum sustainable yield, especially given that harvest volume and value do not always correlate with nutrient provision (Hicks et al. 2019). Thus, policies should focus not only on conserving and rebuilding economically valuable fisheries, but also on sustainably managing nutrient-rich stocks. Such an approach may uncover opportunities to diversify fish production without increasing pressure on existing stocks.

### Govern distribution

Availability, access, and stability are key dimensions of food and nutrition security that can be directly influenced by policy, by linking governance of production with governance of distribution and post-harvest processes. Even though fish is one of the most traded food commodities in the world, information about post-harvest distribution and the kinds of policies that work best to improve distributional outcomes is limited. Rigorous studies on the pathways linking fish to food and nutrition security (direct consumption, income, women’s empowerment) remain scarce (Kawarazuka and Béné 2011). As a result, it is unclear, for example, under what circumstances fish exports detract from food and nutrition security (by exporting essential nutrients from poor coastal populations to wealthy consumers who bid up prices) or enhance food and nutrition security (by bolstering economic development and purchasing power). If fish is sold instead of consumed locally, and less nutritious, processed foods are purchased, then undesirable nutritional outcomes can result (Paddock 2017).

It is crucial to manage distributional dimensions across different food system components, including explicit attention to gender. For example, important policy objectives include protecting fishing access rights for small-scale fishers, addressing power relations in fish value chains that disadvantage small-scale fish workers—many of whom are women—and ensuring that export markets support broad-based development and not just highly capitalized firms. The distribution of capital and property rights to harvest and produce fish is an essential consideration in promoting equitable nutrition and livelihood benefits from fish value chains, especially given the tight links between harvesting and post-harvesting institutions in large- and small-scale commercial fisheries (Asche et al. 2018; Basurto et al. 2020). A gendered approach to policy development is a crucial cross-cutting perspective linking fish production, post-harvest processing and trade, and household nutritional outcomes, as women play important roles in fisheries and aquaculture sectors but are often underrepresented and marginalized in research and policy (Harper et al. 2013). The WorldFish research program on value chains and nutrition is making inroads toward these policy goals through research illuminating effective strategies to enhance the availability, accessibility, and consumption of nutrient-rich, safe fish by poor consumers, particularly women and children.^9^

### Situate fish in food systems framework

Finally, policy makers require decision tools that conceptualize capture fisheries and aquaculture as components of the food systems framework (HLPE 2017, p 26). Recent analyses underscore that co-optimizing human nutrition and sustainability entails a variety of potential synergies and trade-offs among different environmental impacts of food production (e.g. land use, water footprint, greenhouse gas emissions, overfishing) and human health impacts (e.g. provision of diverse, nutritious foods in diets, reducing disease burden from processed foods) (Gephart et al. 2016). For example, reducing the world’s consumption of ASFs is necessary, but alleviating the global burden of micronutrient deficiencies with plant-source foods alone is unlikely. Freshwater is needed to irrigate crops, but water diversion and agricultural run-off can harm inland and coastal fisheries (Jackson 2008; Youn et al. 2014). Demand for feed in animal production, including aquaculture, increases feed prices, creating incentives to innovate and reduce dependency on fish meal and fish oil (Asche and Smith 2018). But these same high feed prices incentivize overfishing in poorly managed fisheries that provide feed inputs. Balancing incentives for innovation in aquaculture and the need to govern capture fisheries effectively illustrate the complementarity of fish as food and conservation perspectives. Relatedly, output in aquaculture can reduce pressure on capture fisheries that compete in the same markets, but only if aquaculture production does not unduly harm the ecosystems on which both depend. A global, multisectoral food systems framework helps to avoid unintended consequences of fish production, for example, by drawing attention to the current and potential contributions of low-trophic aquaculture and thoughtfully sited marine aquaculture that do not tax terrestrial environments through fish feed production or land conversion (Gentry et al. 2017; Belton et al. 2018). The salience of these trade-offs will likely increase as terrestrial food production systems become more stressed by climate change (IPCC 2019). Integrated, multi-sectoral policies that weigh these trade-offs can only emerge within a fish-as-food framework, requiring a better understanding of the connections among fish production and distribution, terrestrial agriculture, and human and planetary health.

## Conclusion

There is growing consensus about the need to recognize the crucial contributions of fish to global food and nutrition security. However, based on our analysis of high-level funding portfolios and policy dialogues, we argue that this consensus is not yet reflected in the sustainable development discourse of powerful international organizations and actors. This is important because discourse—the way that interest groups and stakeholders frame, categorize, value, and study an issue—shapes the range of policies that are conceivable and the kinds of data and knowledge that are generated (Dryzek 2013). How people and institutions imagine the value of fish to society (as a natural resource, a commodity, a food) has powerful implications for governance and the social and ecological impacts thereof.

Fisheries and aquaculture are falling short of their potential to contribute to nutrition and food security. This pattern will continue unless nutrition and food security are explicit policy and funding priorities. For example, as demonstrated by a study in Bangladesh, expanding aquaculture can compensate for stagnated or falling capture fisheries production in terms of volume, but there is no guarantee farmed fish can compensate for the *nutrients* provided to the poor by wild caught fish unless nutrition is an explicit objective (Belton et al. 2014). Global fish harvests may yield sufficient macro- and micronutrients for adjacent coastal populations, but post-harvest dynamics may be such that nutritional deficiencies persist in those areas (Hicks et al. 2019).

Framing fish as food highlights data gaps and the need for new metrics to better understand current and potential nutrient production and how those nutrients flow across a landscape of variable human nutrition needs and deficits, providing food and livelihoods. This new knowledge, in turn, can enable policy to attune capture fisheries and aquaculture governance to enhance nutritional dimensions of production and distribution. Ultimately, it places fish in the broader conversation about the role of food systems in nourishing people and sustaining earth’s ecosystems.

Situating fish within a broader food systems framework highlights the ways in which the importance of fish as food depends upon and can serve to promote conservation and sustainability. Expanding the current ‘sustainable seafood discourse’ widens the scope of relevant environmental issues and diversifies the potential spaces for intervention, including engaging actors (e.g. service/input providers, processors, distributors, etc.) and related processes (e.g., processing efficiency, energy use, food loss and waste, overconsumption of protein) across fish value chains to promote sustainability (Tlusty et al. 2019). Capture fisheries and aquaculture each face distinct environmental challenges, from overfishing and destructive fishing practices in the case of the former, to problems with energy and water use, fish feed, invasive species, pathogens, antibiotic use, and release of nutrients and pollutants in the case of the latter. However, environmental impact varies widely across species and production methods, with some types of capture fisheries and aquaculture providing nutritious ASF at a lower environmental cost than livestock production systems (Hilborn et al. 2018), indicating that with the right policy priorities and actions, environmental and human health synergies are possible.

Positioning fisheries as a vital food source in the context of the UN SDGs and other international efforts to alleviate hunger and malnutrition can provide a strong incentive to invest in sustainable governance, in addition to traditional biodiversity conservation and economic development goals. The food and nutrition security argument is crucial for the many small-scale and developing country fisheries around the world in which the economic costs of improving management would seem to outweigh potential economic returns on investment. Demonstrating the potential of these fisheries to enhance food and nutrition security can encourage governments and international development organizations to invest where they have failed to do so in the past. Aquaculture comprises a diverse set of food production practices, with ample opportunities to implement socially just and environmentally sound policies (Gephart et al. 2020). Both capture fisheries and aquaculture already make crucial contributions to global food and nutrition security. However, the right policies are needed to maintain and enhance these contributions. Here, we promote a “fish as food” discourse by outlining a research and policy road map to prioritize fish as food in development funding priorities and policy dialogues.

## Electronic supplementary material

Below is the link to the electronic supplementary material.

Supplementary material 1 (PDF 720 kb)

## Footnotes

1 We use the term fish to refer to all animal products (including crustaceans, molluscs, cephalopods, and other marine invertebrates) harvested from aquatic systems (including both marine and inland systems).

2 Food and nutrition security exists when all people at all times have physical, social and economic access to food, which is consumed in sufficient quantity and quality to meet their dietary needs and food preferences, and is supported by an environment of adequate sanitation, health services and care, allowing for a healthy and active life (Committee on World Food Security 2012).

3 FAO Food Balance Sheets (http://www.fao.org/faostat/en/#data/FBS/metadata).

4 Visit https://sustainabledevelopment.un.org/ to read the full list of Sustainable Development Goals and their targets and indicators.

5 https://globalnutritionreport.org, accessed August 3, 2018.

6 https://www.gatesfoundation.org/Who-We-Are/Resources-and-Media/Annual-Letters-List, accessed August 3, 2018.

7 See ‘Global Expanded Nutrient Supply Dataverse (GENuS)’: https://dataverse.harvard.edu/dataverse.xhtml?alias=GENuS.

8 Biofortification is the process of increasing micronutrients in the edible portion of plants through plant breeding.

9 https://www.worldfishcenter.org/content/value-chains-and-nutrition.
