# Supplementary material for: Recognize fish as food in policy discourse and development funding
Source: Ambio. 2021 Jan 16;50(5):981–9. doi: 10.1007/s13280-020-01451-4 (PMC7811336; doi:10.1007/s13280-020-01451-4)
Supplement: Supplementary file 1 — Supplementary material 1 (PDF 720 kb) [file 13280_2020_1451_MOESM1_ESM.pdf]

Ambio

Electronic Supplementary Material
This supplementary material has not been peer reviewed

Title: **Recognize fish as food in policy discourse and development funding**

Abigail Bennett, Xavier Basurto, John Virdin, Xinyan Lin, Samantha J Betances, Martin D Smith, Edward H Allison, Barbara Best, Kelly D Brownell, Lisa M Campbell, Christopher D Golden, Elizabeth Havice, Christina C Hicks, Peter J Jacques, Kristin Kleisner, Niels Lindquist, Rafaella Lobo, Grant D Murray, Michelle Nowlin, Pawan G Patil, Douglas N Rader, Stephen E Roady, Shakuntala H Thilsted, Sarah Zoubek

Methods for Figure 2*

*datasets are available upon request to the corresponding author.

World Bank funding commitments data analysis

World Development Bank funding commitment data was accessed through the World Bank's project funding webpage (http://www.projects.worldbank.org/). On the site, the World Bank organizes its projects according to sectors. The "Agriculture" sector includes agricultural funding, forestry funding, and fisheries and agriculture funding. Calculating the year-by-year funding allocation to fisheries and aquaculture as a percent of total agriculture sector funding was a multi-step process.

1. A list of fisheries and aquaculture projects was generated using the key word search function of the project website. A second list of fisheries and aquaculture projects was generated using the subsector filter "fisheries" available on the project website. The reason for this dual approach to finding fisheries funding was that the World Bank's 'fisheries' subsector filter did not seem to identify all fisheries-related projects, and some projects identified in the filter did not include fisheries components, which was apparent upon closer inspection of project titles and documents.
2. Because the two fisheries and aquaculture lists overlapped incompletely, we used an Excel add-on ("Ablebits") to compare the two lists in Excel and eliminate overlap to generate a master list.
3. Because many World Bank projects include multiple subsectors, such as fisheries and crops, we attributed the proportion of funding according to the percentages provided for each such project under the 'details' tab of the project page on the World Bank website.
4. Then, we pulled all forestry projects and assessed their funding percentages indicated on each project's 'details' tab. It was important to identify forestry funding because we presume forestry is largely unrelated to food production. Therefore, we removed this funding from the analysis entirely.
5. Finally, we calculated the year-by-year funding allocation to fisheries and aquaculture as the percent of total agriculture sector funding, excluding forestry.

World Bank project website:
(http://projects.worldbank.org/search?lang=en&searchTerm=&mjsectorcode_exact=AX)

Regional development banks funding commitments data analysis

The approach to regional development bank funding allocations was similar to that used for the World Bank funding data analysis. We downloaded project data as an excel file for all years available from the regional development bank websites. The excel files included information such as project title, year, funding commitment, short description, sector, and subsector. We generated a list of fisheries and forestry projects through keyword search within the excel file. Keyword searches included English terms fish* and aqua* and terms from additional languages where project titles were non-english, (e.g. peche (fish) for the African Development Bank and pesc*/pesq* for the Inter-American Development Bank). Forestry, cotton, and coffee were searched and those projects were removed from the analysis. Finally, project titles were read manually for quality assurance/control. Any projects with ambiguous titles and descriptions were searched on the Bank's website and project documents read to determine if the project included a fisheries or aquaculture component. Then, the year-by-year percent allocation of funding to

fish was calculated. None of the three regional development bank websites included percentages associated with different subsectors, however the incidence of multi-sector projects was much lower than in the World Bank project portfolio. We employed a conservative approach of allocating any project with a fisheries subcomponent entirely to fisheries.

African Development Bank project website: https://www.afdb.org/en/projects-and-operations

Asian Development Bank project website: https://www.adb.org/projects

Inter-American Development Bank project website: https://www.iadb.org/en/projects

Committee on Fisheries reports Quantitative Content Analysis

The methods used were the same as described in detail in Lobo and Jacques (2017). The method of quantitative content analysis (QCA) consisted of various steps, including the pre-processing of the COFI reports to eliminate pages that would inflate certain categories (i.e. the various pages with delegates positions and addresses), and to make sure they are readable to the software. Provalis Wordstat 7.1.14 was used to run the same dictionary created and described in detail in Lobo and Jacques (2017) and further refined in Jacques and Lobo (2018), but with the COFI reports. This dictionary was modified from the dictionary in Bengston, et. al (2004). As explained then, the dictionary went through a series of hand and automated validations, including factor analysis to see proximate and semantic relationship, and inter-coder reliability tests to ensure the rules for making the categories were reliable.

This time around, we once again used the Keywords in Context (KWIC) recall to validate the dictionary was working as intended, and used the Wordstat thesaurus to ensure related words were not missed.

Categories

Economic

This category encompasses anything related to economic/utilitarian principles, such as trade, production, market, commerce, exploitation, etc.

Governance

Anything related to governance, government, legal and formal systems of authority and control. For example: regulation, management, compliance, regime, jurisdiction, etc.

Social Values

This category evokes social values as understood in the broader social sciences literature, such as equity, justice, artisanal, poverty, etc.

Environmental/Ecological

A broad category that encompasses everything related to life support systems, such as ecosystem, climate, ecology, acidification, wildlife, etc.

Science

Words related to the scientific endeavor, such as data, research, sample, evidence, model, etc.

Food

Categories of word related to a food discourse, such as diet, protein, nutrition, subsistence, sustenance, etc.

Specific Fisheries

Mentions of specific important fisheries, such as salmon, tuna, sardines, or even protected species like whales and turtles.

Sustainability

Discourses that relate to sustainability, sustainable development, principles of sustainability, as analyzed in detail in Jacques and Lobo (2018).
